# Three-dimensional multiway power dividers based on transformation optics

**DOI:** 10.1038/srep24495

**Published:** 2016-04-19

**Authors:** Yong-Le Wu, Zheng Zhuang, Li Deng, Yuan-An Liu

**Affiliations:** 1 Beijing Key Laboratory of Work Safety Intelligent Monitoring, School of Electronic Engineering, Beijing University of Posts and Telecommunications, P.O. Box. 282, 100876, Beijing, China; 2 Beijing Key Laboratory of Network System Architecture and Convergence, School of Information and Communication Engineering, Beijing University of Posts and Telecommunications, P.O. Box. 282, 100876, Beijing, China

## Abstract

The two-dimensional (2D) or three-dimensional (3D) multiway power dividers based on transformation optical theory are proposed in this paper. It comprises of several nonisotropic mediums and one isotropic medium without any lumped and distributed elements. By using finite embedded coordinate transformations, the incident beam can be split and bent arbitrarily in order to achieve effective power division and transmission. In addition, the location of the split point can be employed to obtain unequal power dividers. Finally, several typical examples of the generalized power divider without limitation in 3D space are performed, which shows that the proposed power divider can implement required functions with arbitrary power division and arbitrary transmission paths. The excellent simulated results verify the novel design method for power dividers.

Power dividers are widely applied in microwave and wireless communication systems, especially for the feeding network of antenna arrays and power amplifiers. The original power divider composed of quarter-wavelength transmission lines is presented by Wilkinson in^1^. Over the past several years, extensive researches have been developed on the conventional Wilkinson power divider with various good performances^2^,^3^,^4^,^5^. A generalized Wilkinson power divider with multifunction including multiway transmission, dual-band operation, and unequal power division is designed by incorporating the extended dual-band power divider cell, recombinant structure, and the two-section dual-frequency transformers^2^. Two-section coupled lines and two isolation resistors are utilized to realize compact circuit structure and good isolation^3^. In addition, a single-section coupled line is demonstrated for arbitrary complex input and output terminated impedances^4^. Furthermore, the impedance-transforming coupled-line Wilkinson power divider can be constructed using three additional extended transmission lines with enhanced physical output isolation^5^. Although microstrip-based power dividers have been widely investigated, several inevitable disadvantages of the low power mode and large insertion loss operating at high frequency still exist, leading the quasi-planar power dividers using coaxial waveguide^6^ and reconfigurable power dividers based on switch elements^7^,^8^.

However, aforementioned power dividers generally need tedious design methods such as rigorous even- and odd-mode analysis or transmission theory, resulting in complicated design processes and circuit structures. Fortunately, transformation optics brings in a brand-new concept to manipulate power flow^9^,^10^, which provides a new direction for designing a power divider with various excellent performances. In recent years, transformation optics is mainly applied in optical cloaks^11^, waveguide bends and corners^12^, illusion optics devices^13^, beam bends and expanders^14^. Soon after, some researches start to extend to 3D-transformation optical fields^15^,^16^.

In this paper, a 3D multiway power divider with various properties including arbitrary power division and arbitrary transmission paths is proposed based on transformation optical theory. The multiway power divider is constructed by an isotropic medium serving as incident field and several nonisotropic mediums acting as exited field. By introducing coordinate transformations, the incident beam can transmit along arbitrary paths and the power of incident beam can be distributed by controlling the location of the beam split. Moreover, it is worth noting that transformation optics is unlimited for any operating frequency. Compared with conventional design methods of power dividers, the proposed one can present a simpler design process and structure on the basis of achieving the above required properties, solving the main problem of complicated design processes and circuit structures in conventional power dividers for multiway design. Hence, the advantages of the proposed 3D multiway power divider based on transformation optics can be summarized as follows: 1) simple design methods and structure, 2) multiway arbitrary transmission paths, 3) arbitrary power division, and 4) minimization for 3D multiway power dividers.

## Methods

### Theoretical design

The relative permittivity *ε*_ij_’ and permeability *μ*_ij_’ of the transformation optical medium can be defined by the following expressions^12^:


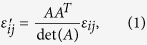


and


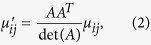


where *A*^T^ indicates the transposed matrix of the Jacobi matrix of the transformation *A* which can be calculated as


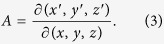


Therefore, if giving an adaptive coordinate transformation, the relative permittivity and permeability of transformation optical beam bend can be obtained easily according to equations (1, 2, 3). Furthermore, when the incident beam accesses to different transformation optical mediums, it would be split into two beams with different power determined by the location of the split point. Finally, a 2D/3D power divider can be designed by the above transformation optical theory.

### Numerical calculation

The simulated results are obtained by using the multiphysics simulation tool based on the finite element method (FEM). The corresponding power calculations are realized via line integral (2D) or surface integral (3D) in ports.

## Results

### 2D two-way power divider (TWPD)

**Figure 1:**
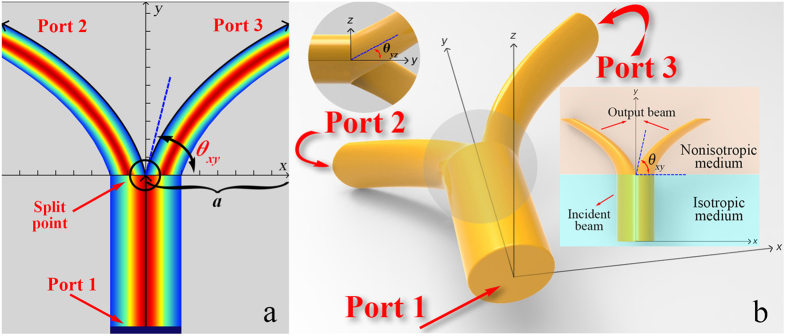
Ideal coordinate transformations. (**a**) Ideal coordinate transforming paths of 2D-TWPD. θ_xy_ is determined by the parameter *a*. The port boundary is used to serve as an excitation source of electric field. (**b**) Ideal coordinate transforming paths of 3D-TWPD. θ_xy_ and θ_yz_ are determined by parameters *a* and *k*, respectively. It should be noted that the Gaussian beam excited at Port 1 acts as the incident beam.

The proposed 2D TWPD is composed of one isotropic medium and two different nonisotropic mediums. In general, the relative permittivity and permeability of isotropic medium as incident area are set as 1. A possible coordinate transformation for a beam bend in nonisotropic mediums can be given by


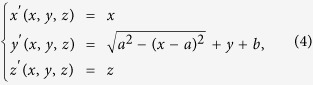


where *a* and *b* denote the central coordinate of circle for a beam bend, respectively. Fig. 1a illustrates the ideal coordinate transformation of 2D TWPD. Based on equations (1)–(4), we can obtain


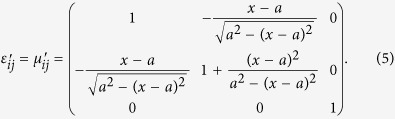


**Figure 2:**
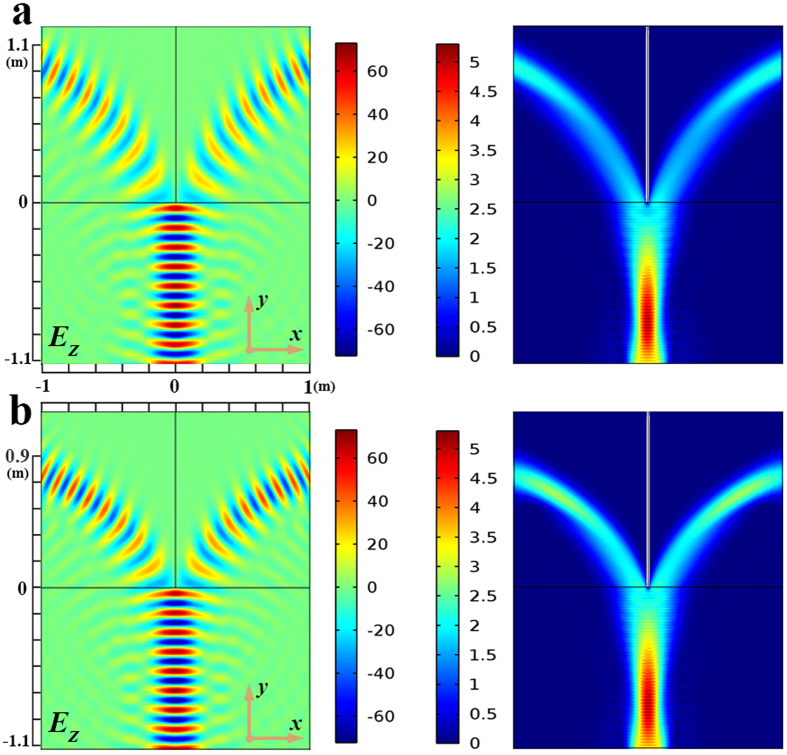
Results of the proposed 2D-TWPD. (**a**) Electric field and power flow distributions of the 2D-TWPD with *a* = ±1 m. The corresponding z-component of the electric field operating at 2.3 GHz is illustrated. Meanwhile, the power division can be clearly seen in different transformation optical mediums. (**b**) Electric field and power flow distributions of the 2D-TWPD with *a* = ±0.7 m. The corresponding z-component of the electric field operating at 2.3 GHz is illustrated. Compared with Fig. 2a, different transmission paths can be controlled by adjusting the value of *a*.

As can be clearly seen from equation (5), the nonisotropic material parameters are only dependent on *a*. Hence, the transmission paths of TWPD can be controlled by *a* freely. To confirm the validity of this method, we carry out full wave simulations using the multiphysics simulation tool as a finite element solver. In all numerical calculations, the transformation optics is contained within a rectangular area with perfectly matched layer boundaries. The operating frequency of the wave used in these simulations is 2.3 GHz. Besides, the port boundary is used to act as an excitation source of electric field with input power of 1 W. Fig. 2 shows the spatial distribution of the electric field and power flow of 2D equal TWPD with different transmission paths, respectively. In Fig. 2a, the nonisotropic material parameters are determined by *a* = ±1 m while the corresponding parameters are calculated for *a* = ±0.7 m in Fig. 2b. Meanwhile, the input power in Fig. 2a is calculated by line integral as 0.92 W, whereas the power of two output ports is both 0.44 W, respectively. Similarly, the input power in Fig. 2b can be obtained by line integral as 0.96 W while the power of two output ports is both 0.46 W. In addition, the sizes of 2D-TWPDs are depicted as Fig. 2. It should be noted that the lengths along x are both set as 1 m for comparing the different transmission paths with different values of *a*, whereas the length along y of incident field may not be limited strictly. Thus, the sizes of the proposed 2D-TWPDs except for the incident field are about 2.2 m^2^ (2 × 1.1 m^2^) and 1.8 m^2^ (2 × 0.9 m^2^), respectively. It is worth noting that the size of the proposed 2D-TWPD is very large at the operation frequency of 2.3 GHz while the main purpose for introducing the design example of the 2D-TWPD is to more clearly explain the design details from the transformation-optics view for the proposed 3D-MWPD. Size minimization is only for the 3D-MWPD case.

**Figure 3:**
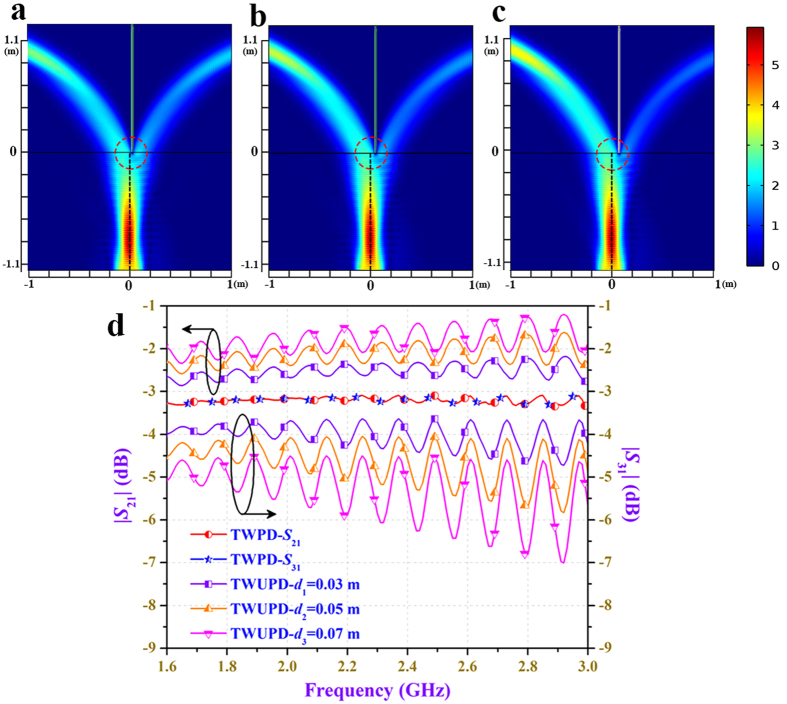
Results of the proposed 2D-TWUPD. (**a**) Power flow distributions of the 2D-TWUPD with *a* = ±1 m, d = 0.03 m. (**b**) Power flow distributions of the 2D-TWUPD with *a* = ±1 m, *d* = 0.05 m. (**c**) Power flow distributions of the 2D-TWUPD with *a* = ±1 m, *d* = 0.07 m. The operating frequency of all simulations is 2.3 GHz. By changing the value of offset distance, unequal power division can be implemented in two output transmission paths. (**d**) S-parameters of two-way power dividers with different power division ratios. It can be seen that power division changes with offset distance variation. The power division ratio at 2.3 GHz is 0.65 with *d* = 0.03 m and the power division ratios at 2.3 GHz are 0.49 and 0.37 when *d* = 0.05 m and *d* = 0.07 m, respectively.

In order to further achieve unequal power division, the adaptive location of the split point needed to be chosen in two different transmission paths. In other words, the location of the split point will be off the center of the incident area. The corresponding material parameters need to be rewritten as


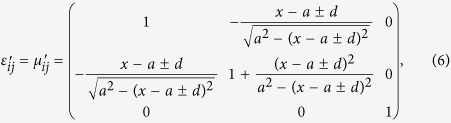


where *d* represents the offset distance of the split point and ‘±’ denotes left and right offset direction. The power flows of two-way unequal power dividers (TWUPDs) with different power division ratios are depicted in Fig. 3 and the corresponding *S*-parameters transformed from power division ratios are shown in Fig. 3d. From aforementioned simulated results, when the incident beam excited in the input port accesses to the two transformation optical mediums with different material parameters, it is split into two beams of different directions with lower power. Therefore, using transformation optics can implement the required function with power division and then the power in output ports can be controlled independently by the location of the split point. It is worth noting that the directions of the two split beams can be determined by the parameter *a* according to the demands in practical applications.

### 3D multiway power divider (MWPD)

In practical applications, 3D devices generally need to be manufactured instead of 2D devices. In addition, it is easier to achieve multi-way transmission in 3D space comparing with 2D space. For the purpose of satisfying the above demands, this paper also introduces an effective coordinate transformation shown in Fig. 1b for constructing a 3D MWPD. The corresponding coordinate transformations can be defined as


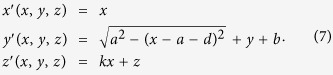


And then according to equations (1, 2, 3) and (7), the material parameters of 3D transformation optical space are deduced as


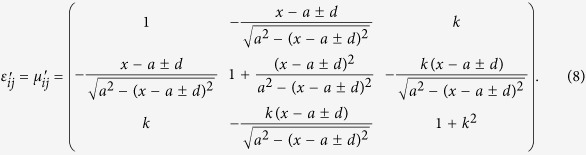


**Figure 4:**
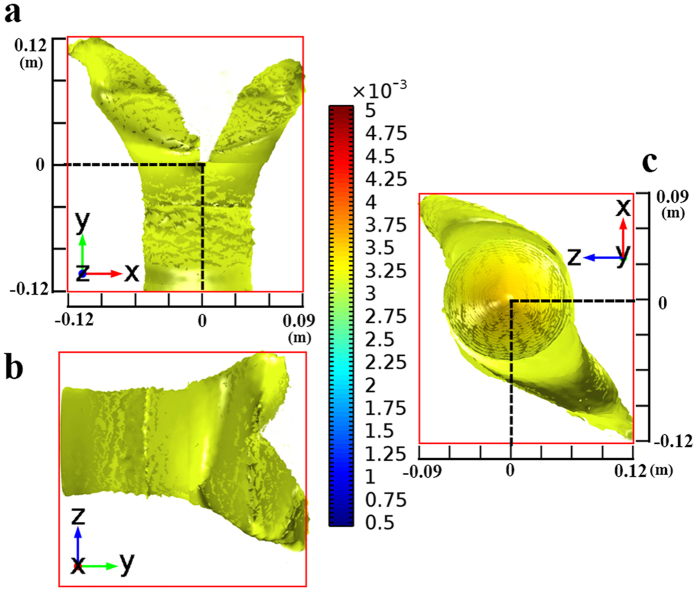
Results of the proposed 3D-TWUPD. (**a**) Power flow distributions in x-y plane. (**b**) Power flow distributions in y-z plane. (**c**) Power flow distributions in z-x plane. The value of input power calculated by surface integral is 12.7 μW while the values of output power are calculated as 6.71 μW and 5.90 μW, respectively.

**Figure 5:**
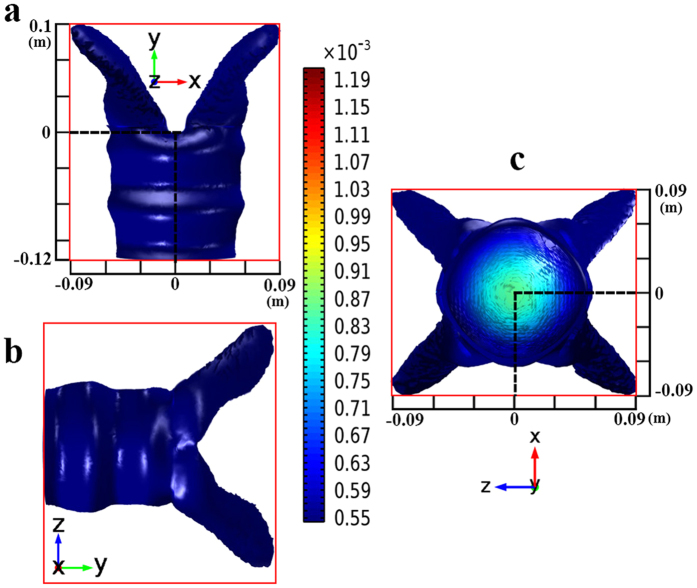
Results of the proposed 3D-FWEPD. (**a**) Power flow distributions in x-y plane. (**b**) Power flow distributions in y-z plane. (**c**) Power flow distributions in z-x plane. The value of input power calculated by surface integral is 16.9 μW, whereas the values of four output powers are equal to 4.53 μW.

As shown in equation (8), the transmission paths in 3D space can be tuned by the parameters *a*, *d*, and *k*. To validate the proposed design methods of 3D MWPD, we adopt full wave simulations based on the multiphysics simulation tool as a finite element solver. The corresponding simulated examples operated at 3 GHz are illustrated in Figs. 4 and 5. Hereinto, Fig. 4 shows the isosurface for power flows of a 3D two-way unequal power divider (TWUPD) with *a* = ±0.12 m, *d* = 0.004 m, and *k* = 1, whereas the isosurface for power flows of a 3D four-way equal power divider (FWEPD) is shown in Fig. 5 with *a* = ±0.12 m, *d* = 0, and *k* = ±1. For the sake of showing the function with power division, the values of power can be calculated by surface integral in input and output ports. Besides, the sizes of 3D-TWUPD and 3D-FWEPD except for the incident field are about 0.005292 m^3^ (0.21 × 0.12 × 0.21 m^3^) and 0.00324 m^3^ (0.18 × 0.1 × 0.18 m^3^), respectively. Note that the length along z can be controlled by the parameter *k* according to practical applications. Similarly, the 3D MWPD can be designed by the transformation optical theory, with functions of arbitrary multiway transmission paths and arbitrary power division.

## Discussion

To the authors’ best knowledge, for the first time transformation optical design of 3D multiway power dividers is presented. At present, a great deal of investigations for power dividers based on microstrip^1^,^2^,^3^,^4^,^5^,^7^,^8^ or waveguide^6^ generally have complicated structures or design processes in order to implement the required functions with unequal power division and multi-way transmission. Fortunately, transformation optical design theory can not only fulfill the requirement of power division but also make the best of 3D space to implement multi-way transmission, which shows the potential of minimization. Although the size of the proposed 2D-TWPD is very large at the operation frequency of 2.3 GHz, the occupied space for 3D-MWPD becomes much smaller than the size of the 2D-TWPD aforementioned both for the dielectric material of air, indicating an obvious size-minimization function for 3D-MWPDs. Furthermore, because 3D-MWPD based on transformation optical theory is achieved inside the medium without any additional area, the occupied sizes of two-way transmission and four-way transmission in 3D space are almost identical as shown in Figs. 4 and 5. Hence, 3D-MWPDs based on transformation optics have no use for adding additional design space and complicated circuit structure compared with conventional multiway power dividers, further demonstrating the function of minimization for designing multi-way transmission. To sum up, the presented idea in this paper provides a novel and effective design direction for complex multifunction power dividers, especially for multi-way transmission.

## Additional Information

**How to cite this article**: Wu, Y.-L. *et al.* Three-dimensional multiway power dividers based on transformation optics. *Sci. Rep.* **6**, 24495; doi: 10.1038/srep24495 (2016).
